# Supplementary material for: The SILKIE (Skin graftIng Low friKtIon Environment) study: a non-randomised proof-of-concept and feasibility study on the impact of low-friction nursing environment on skin grafting success rates in adult and paediatric burns
Source: BMJ Open. 2018 Jun 14;8(6):e021886. doi: 10.1136/bmjopen-2018-021886 (PMC6009614; doi:10.1136/bmjopen-2018-021886)
Supplement: Supplementary file 1 [file bmjopen-2018-021886supp001.pdf]

Appendix 1. Participant flow to questionnaires and interviews looking at resource use and patient views of the low friction intervention.

334 screened and assessed for eligibility for low friction nursing over 15 months

Not eligible: n=203 (61%)

- Day case only (no overnight stay): 51 (25%)
- Ventilated or on inotropes: 4 (2%)
- VAC dressings were used to maintain grafts: 3 (1.5%)
- Allograft: 6 (3%)
- Not burns: 19 (9%)
- Not skin grafted/surgical plan altered: 24 (12%)
- Missed by research/ward staff: 5 (2%)
- Ineligibility details unavailable: 91 (45%)

131 eligible for low friction nursing cohort (39%)

Not approached for consent to questionnaires/interview: n = 59 (45%)

- No research staff availability: 15 (25%)
- Patient mental health issues: 13 (22%)
- Clinical staff request: 8 (14%)
- Originally day case – consent not sought 5 (8%)
- Non-English speaking: 2 (3%)
- Child protection issues: 2 (3%)
- Parents injured at same time: 1 (2%)
- Other: 13 (22%)

72 patients or parents of patients approached for consent (55%)

Did not consent: n = 20 (28%)

- No research staff availability: 1 (5%)
- Patient mental health issues: 1 (5%)
- Literacy issues: 2 (10%)
- High levels of stress around injury: 4 (20%)
- Patient/carer not interested: 2 (10%)
- No reason given: 6 (30%)
- Other: 4 (20%)

52 (72%) consented to questionnaires and 49 (68%) consented to interview

48 completed at least one questionnaire (92%)
32 participants completed all three questionnaires (62%)
17 interviews carried out (35%)
