# Supplementary material for: The SILKIE (Skin graftIng Low friKtIon Environment) study: a non-randomised proof-of-concept and feasibility study on the impact of low-friction nursing environment on skin grafting success rates in adult and paediatric burns
Source: BMJ Open. 2018 Jun 14;8(6):e021886. doi: 10.1136/bmjopen-2018-021886 (PMC6009614; doi:10.1136/bmjopen-2018-021886)
Supplement: Author's manuscript [file bmjopen-2018-021886.draft_revisions.pdf]

BMJ Open

BMJ Open is committed to open peer review. As part of this commitment we make the peer review history of every article we publish publicly available.

When an article is published we post the peer reviewers' comments and the authors' responses online. We also post the versions of the paper that were used during peer review. These are the versions that the peer review comments apply to.

The versions of the paper that follow are the versions that were submitted during the peer review process. They are not the versions of record or the final published versions. They should not be cited or distributed as the published version of this manuscript.

BMJ Open is an open access journal and the full, final, typeset and author-corrected version of record of the manuscript is available on our site with no access controls, subscription charges or pay-per-view fees (http://bmjopen.bmj.com).

If you have any questions on BMJ Open's open peer review process please email info.bmjopen@bmj.com

BMJ Open

The SILKIE (Skin graftIng Low friKtIon Environment) study: a non-randomised proof-of-concept and feasibility study on the impact of low friction nursing environment on skin grafting success rates in adult and paediatric burns.

Journal:	*BMJ Open*
Manuscript ID	bmjopen-2018-021886
Article Type:	Research
Date Submitted by the Author:	30-Jan-2018
Complete List of Authors:	Hollen, Linda; The Scar Free Foundation Centre for Children's Burn Research, University Hospitals Bristol NHS Foundation Trust; Bristol Medical School, University of Bristol, Centre for Child and Adolescent Health<br>Greenwood, Rosemary; University Hospitals Bristol NHS Foundation Trust, Research and Design Service<br>Kandiyali, Rebecca; The Scar Free Foundation Centre for Children's Burn Research, University Hospitals Bristol NHS Foundation Trust; Bristol Medical School, University of Bristol, Centre for Child and Adolescent Health<br>Ingram, Jenny; Bristol Medical School, University of Bristol, Centre for Child and Adolescent Health<br>Foy, Chris; Gloucester RDSU<br>George, Susan; The Scar Free Foundation Centre for Children's Burn Research, University Hospitals Bristol NHS Foundation Trust<br>Mulligan, Sandra`; The Scar Free Foundation Centre for Children's Burn Research, University Hospitals Bristol NHS Foundation Trust<br>Spickett-Jones, Francesca; The Scar Free Foundation Centre for Children's Burn Research, University Hospitals Bristol NHS Foundation Trust<br>Booth, Simon; Queen Victoria Hospital NHS Foundation Trust, McIndoe Burn Centre<br>Sack, Anthony ; North Bristol NHS Trust, Southmead Hospital<br>Emond, Alan; The Scar Free Foundation Centre for Children's Burn Research, University Hospitals Bristol NHS Foundation Trust; Bristol Medical School, University of Bristol, Centre for Child and Adolescent Health<br>Young, Amber; The Scar Free Foundation Centre for Children's Burn Research, University Hospitals Bristol NHS Foundation Trust
Keywords:	skin grafting, low friction, burn injuries, graft loss, burns

SCHOLARONE™
Manuscripts

The SILKIE (Skin graftIng Low friKtIon Environment) study: a non-randomised proof-of-concept and feasibility study on the impact of low friction nursing environment on skin grafting success rates in adult and paediatric burns.

Linda Hollén[1,2], Rosemary Greenwood[3], Rebecca Kandiyali[1,2], Jenny Ingram[2], Chris Foy[4], Susan George[1], Sandra Mulligan[1], Spickett-Jones Francesca[1], Booth Simon[5], Sack Anthony[6], Alan Emond[1,2], Amber Young[1]

[1] The Scar Free Foundation Centre for Children's Burn Research, Bristol Royal Hospital for Children, University Hospitals Bristol NHS Foundation Trust, Bristol, UK.

[2] Centre for Child and Adolescent Health, Bristol Medical School, University of Bristol, Oakfield House, Oakfield Grove, Bristol, UK.

[3] Department of Research and Innovation, University of Hospitals Bristol NHS Foundation Trust, Bristol, UK.

[4] Gloucestershire Research Support Service, Gloucestershire Hospitals NHS Foundation Trust, Gloucester, UK.

[5] McIndoe Burns Centre Queen Victoria Hospital NHS foundation Trust, East Grinstead, UK.

[6] North Bristol NHS Trust, Southmead Hospital, UK.

Correspondence to: Dr Amber Young, email: amber.young1@nhs.net

For peer review only

ABSTRACT

Objectives: To evaluate the impact of low friction (LF) bedding on graft loss in an acute burn care setting, and to examine the feasibility, and costs of using LF bedding compared to standard care.

Design: Proof of concept before and after study with feasibility of delivering the intervention.

Setting: Three burns services within two UK hospital trusts.

Participants: Inclusion criteria were patients older than four weeks, who received a skin graft after burn injury and were admitted overnight. The comparator cohort were eligible patients admitted in a 12-month period before the intervention.

Intervention: Introduction of LF sheets and pillowcases during a 15-month period.

Outcome measures: For proof of concept, the LF and comparator cohorts were compared in terms of number of re-grafting operations (primary), percentage graft loss, average hospital length of stay (LoS), and LoS cost (secondary). Feasibility outcomes were practicality and safety of using LF bedding.

Results: 131 patients were eligible for the LF cohort and 90 patients for the comparator cohort. Although the primary outcome of the proportion needing re-grafting was halved in the LF cohort, the confidence interval crossed 1 (OR [95% CI]: 0.56 [0.16-1.88]). Partial graft loss (any loss) was significantly reduced in the LF cohort (0.27 [0.14-0.51]). Average inpatient LoS was no different between the two cohorts (difference in median days [95% CI]: 0 [-2,1]), and the estimated difference in LoS cost was £-1,139 [-4,829 to 2,551]. Practical issues were easily resolved, and no safety incidents occurred while patients were nursed on LF bedding.

Conclusions: LF bedding is safe to use in burned patients with skin grafts and we have shown proof of concept for the intervention. Further economic modelling is required to see if an appropriately powered randomised control trial would be worthwhile or if roll out across the NHS is justified.

Trial registration: ISRCTN82599687

Keywords: skin grafting, low friction, burn injuries, graft loss, burns

For peer review only

Article Summary

Strengths and limitations of this study

Strengths

- A multi-centre study confirming the feasibility, practicality and safety of using low friction bedding in children and adults with skin grafts.
- Proof of concept that graft loss could be lower in a low friction nursing environment has been demonstrated.

Limitations

- A before and after design restricted the strength of evidence of the effectiveness of the intervention.
- Causality of using a low friction environment for preventing graft loss could not be demonstrated.
- Lack of standardised reporting of partial graft loss limited conclusions of the benefit of the low friction environment.

For peer review only

Contributors: LH performed the analysis of data, contributed to development of methods, interpretation of results, and to the drafting and writing of the manuscript. RG led on development of methods, drafted the research protocol, interpretation of results and to critical reviewing of the manuscript. RK performed the costing analysis, contributed to development of methods, interpretation of results and to critical reviewing of the manuscript. JI contributed to development of qualitative methods, interpretation of results and critical reviewing of the manuscript. CF contributed advice on the costing analysis, contributed to development of methods, interpretation of results and to critical reviewing of the manuscript. SG, SM, FSJ, SB and AS collaborated to develop and lead the intervention, contributed to acquisition of data, interpretation of results and to critical reviewing of the manuscript. AE contributed to development of methods, interpretation of results, consensus meetings and to critical reviewing of the manuscript. AY conceived and led the study and initiated development of the intervention, contributed to development of methods, interpretation of results and to critical reviewing of the manuscript. All authors contributed to and approved the final version and agree to be accountable for all aspects of the work.

Funding: This work was supported by the National Institute for Health Research (NIHR) under its Research for Patient Benefit (RfPB) Programme (Grant Reference No. PB-PG-0214-33003). The views expressed are those of the authors and not necessarily those of the NHS, the NIHR or the Department of Health.

Competing interests: None declared.

Ethics approval: This study had the ethical approval from Wales Research Ethics Committee4 Wrexham (REC reference: 15/WA/0156).

Provenance and peer review: Not commissioned; externally peer reviewed.

Data sharing statement: No additional data available.

For peer review only

INTRODUCTION

Achieving wound closure early after burn injury results in improved survival, better cosmetic outcomes and shorter lengths of hospital stay.[1] To assist wound healing, surgical approaches with early tangential excision and wound closure using skin grafting, are increasingly being applied to deep dermal or full thickness burns.[2,3] Skin grafts are also used in partial thickness burns failing to heal within two to three weeks.[4] There are roughly 1,000 skin grafts undertaken in burns patients annually in England and Wales; 75% undertaken in adults and 25% in children.[5] Integration of grafts to the wound requires vascular and lymphatic revascularization and re-innervation.[6] Healing and 'take' depend upon a well-vascularized clean recipient site, close apposition of the graft to the wound bed and appropriate immobilization of the graft to allow development of new vessels.[7] Sub-optimal take (full or partial graft loss) will delay healing, increase hospital stay, and may also require repeat surgery with increased pain and the potential for infection and increased scar formation with poor cosmetic outcomes.[8,9] These suboptimal outcomes cause distress to patients and impact negatively on physical, social, emotional and economic aspects of their life.[10,11]

Loss of the graft typically occurs during the first few days and may be due to loss of contact with the wound bed due to haematoma, infection or shear.[7,8] It is thought that friction between the dressing and the environment can cause a shearing stress on newly transplanted skin cells and detachment from the wound bed. An even distribution of constant pressure on the graft is therefore likely to increase the chances of successful take.[12] However, maintaining stability and attachment of the graft to the wound-bed can be difficult especially in mobile or semi-mobile patients including children.[13] Low friction (LF) products have been shown to be clinically and cost effective in the prevention of skin breakdown in other at-risk patients[14], but not in patients with burns. LF products are currently in use in the National Health Service

For peer review only

(NHS) in England for premature newborn babies, elderly patients and patients with neurological conditions to prevent pressure ulcers.[15]

We undertook this study using a before and after design to investigate whether a LF nursing environment, using bed sheets and pillow cases (LF [intervention] cohort), could improve skin grafting success rates in adult and paediatric patients with burns compared to standard care using normal bed sheets and pillow cases (comparator cohort). The main aims of this study were: (i) to assess primary and secondary outcome measures around graft loss to provide proof of concept for the use of this technology, (ii) to examine the feasibility of our outcome measures and the practicality and safety of using a LF nursing environment in an acute burn care setting. We also assessed costs associated with the intervention (the purchase, maintenance and laundry of LF items; staff training in the use of LF items) and compared the two cohorts in terms of costs associated with inpatient length of stay (LoS).

METHODS

Study design

We used a non-randomised multicentre study to investigate the feasibility of our intervention. As part of this design, we undertook a before-and-after notes review comparing two cohorts: a low friction (LF) cohort (intervention) and a comparator cohort (standard care), to provide proof of concept through the confidence intervals around effect size.

For peer review only

Participants and data

For both the LF and comparator cohort, patients of more than four weeks of age with burns who received a skin graft and were admitted overnight were assessed for inclusion. Patients were excluded if they were on a ventilator, needing inotropic support or required a Vacuum Assisted Closure (VAC) dressing. Bedding was the only difference between standard care and the LF nursing environment in the form of low friction sheets, cot sheets and pillowcases. Standardised operating procedures were written for the use of these materials both in the bed, in chairs and on equipment used to elevate the wounds. The sheets used were already on the market and available to the NHS.[15] The sheets are made from a synthetic fabric similar to parachute silk, but washable at high temperatures and re-usable. The ultra-low friction fabrics move freely and smoothly over patients' skin and the underlying surface. The sheets conformed to hospital infection control protocols. For the intervention, we expected a three-month adjustment period, after which the standardised operating procedures around LF environment would be finalised. However, as no changes were made during this time, 15 months of data were collected after the introduction of the LF environment. In participating units, the LF environment became standard care, allowing all eligible patients to be included in the intervention cohort. Clinicians could withdraw patients from the LF nursing if it was felt not to be in the best interest of their patient; however, the patient remained in the cohort to prevent biasing the comparison with the comparator cohort (intention to treat analysis). The comparator cohort data were collected for patients from a 12-month period prior to the introduction of LF bedding. Comparator patients were identified from theatre record books, the national burn injury database (iBID)[11] and Trust information systems.

Data were collected in three burns services within two hospital trusts; one serving an adult population, one a paediatric population and the third both adult and paediatric patients.

For peer review only

The same case report form (CRF) was used in both cohorts. Patients in the LF cohort were invited, by the site research nurse, to consent to complete questionnaires on quality of life and resource use and to take part in a telephone interview within two weeks of being discharged from hospital to explore their views of the LF bedding. This study had the ethical approval from Wales Research Ethics Committee4 Wrexham (REC reference: 15/WA/0156).

Outcomes

For peer review only

Primary proof-of-concept outcome

The primary outcome was skin graft failure rate, as assessed by the proportion of patients who underwent re-grafting surgery.

Secondary proof-of-concept outcomes

We assessed two secondary outcomes: percentage graft loss and length of hospital stay. The method of recording percentage graft loss varied between centres but was recorded as either the % graft failure, ≥ 90% healed or ≥ 95% healed. For the purposes of estimating % graft loss, we assumed that a graft that was 90% or 95% healed had less than 10% or 5% loss respectively. Length of hospital stay (LoS) was recorded as the first inpatient LoS (total and per % body surface area [BSA]) and total burn service LoS including repeat admissions.

Feasibility outcomes

We assessed two feasibility outcomes (recorded only in the LF cohort): practicality and safety. The practicality outcome was assessed through staff focus groups or telephone interviews, where any difficulties with bedding use on the wards, laundry problems, and issues with absorbency and staining were discussed. In terms of safety outcomes, we

collected data on falls or slips and wound infection. Formal risk assessments were carried out as required by each site for manual handling, tissue viability and infection control. The site leads received all safety incident reports for the designated clinical areas in “real time”. Background data regarding the numbers of safety incidents were also known to the site leads. To minimise the risk of falls and prevent slipping, the single bed sheets have a low friction sleeping surface with standard sheeting material around the edge of the bed. Further safety improvements included using neonatal cot sheets as shawls to cradle small children instead of full sized sheets to reduce tripping hazards, and the provision of additional pillow cases to facilitate use on equipment rather than full sized sheets. We used two measures of infection: presence of surgical site infections and wound infection as assessed through wound swab results.

Expected sample size

The overall recruitment figure to the LF cohort was dependent on the number of patients who presented with burn injuries requiring skin grafting and an overnight stay at the three study sites during the recruitment period. This was estimated before study start from the national burn database (iBID).[11] A figure of 200 over the recruitment period was initially predicted based on data from the previous five years. After detailed discussion with the database chair however, it became apparent that the data provided from the database were for individual graft events, not patients, i.e. 24% of grafts were multiple procedures in single patients. Expected recruitment numbers were therefore revised to 75% of 200, i.e. 150 patients.

Missing data/data quality

All data were anonymised and entered at each site onto a REDCap (Research Electronic Data Capture) database.[16] Initially, all sites used double data entry for input to REDCap and data

For peer review only - http://bmjopen.bmj.com/site/about/guidelines.xhtml

were checked using the REDCap data comparison facility. Where there were discrepancies, the CRF was checked and the REDCap record amended to contain only correct data. A set of rules was agreed by the management team and applied to subsequent data entry which reduced the error rate and all sites moved to single data entry. Missing data and error checking was continued throughout the study by checking all REDCap records against the CRF for every participant.

Statistical Analyses

Analyses were by intention to treat and performed in Stata v.14.[17] We compared the two cohorts at admission for several demographic and patient characteristic variables: centre admitted to, ethnicity, age, TBSA (total body surface area), gender, burn type, body location burnt, comorbidity present, ASA (American Society of Anesthesiology) grade (physical status classification)[18], body weight, peri-operative antibiotics given, and time to first grafting procedure. Summary statistics used were proportions for categorical variables, and medians and inter quartile range (IQR) or means and standard deviations (SD) for continuous variables. We used two-sample proportion tests to compare the proportion of all categorical demographic and patient characteristic and outcome variables in the two cohorts. Odds ratios and 95% confidence intervals (CI) are reported as a measure of effect size for differences in primary and secondary graft loss outcomes. For LoS outcomes, differences in medians and means with 95% CI are reported.

Economic analyses

The economic work sought to provide information (mean costs and confidence intervals, where appropriate) on the acute health care resource use and costs associated with the new technology, with resources being valued using hospital price information, and national

For peer review only

sources of unit costs for staff time and bed days.[19,20] Resource use data collected focussed on: i. the additional costs associated with the intervention (i.e. the purchase, maintenance and laundry of low friction items; staff training in the use of low friction items); data collection on this element was limited to the LF cohort alone and ii. before and after comparison of inpatient LoS. In our analysis based on reference costs[20], we multiplied the cost of an "inlier" bed day by LoS to estimate inpatient care costs at the level of the patient. To account for the skew in our cost data, we used non-parametric bootstrap methods to construct the confidence interval around the incremental inpatient bed day cost.[21]

Patient and public involvement

Patients and the public have been actively involved at all stages of this study. Early work was undertaken before study start to understand the views of patients with burns undergoing skin grafts. Parent and staff views on the sheet material were also sought ahead of the study at Bristol Royal Hospital for Children and further views were asked during a public engagement event midway through the study. Eleven children (age range 11-17yrs) were involved in developing age-appropriate patient consent and information literature through an established young people's advisory group. Patient and public views on further study patient documentation such as adult information sheet, parent information sheet and the consent forms, were obtained from individuals who contacted the Burns Research Centre in Bristol via the 'People in Research' NIHR website. Four members of the public also read and edited the adult patient documentation, so that the purpose of the project was clearer. Feedback on research design and outcome measures obtained was incorporated into the final study documents. Ms Pam Warren, Ambassador for the Scar Free Foundation and burns survivor, read and edited the study protocol and advised regarding patient and public input. She was also involved in key decision-making through her membership and attendance in person at

the steering group meetings. Patients were not actively involved in the recruitment to and conduct of the study. Following publication of results, we will disseminate to burn patient groups and the public through plain English written documents.

RESULTS

Participant flow, recruitment and compliance

Recruitment to the low friction (LF) cohort started on 6th October 2015 and was completed on 31st December 2016 – a 65-week period. Of the 334 patients screened for eligibility in the LF cohort, 131 (39%) were eligible for low friction nursing. For the comparator cohort, 90 eligible patients were identified across all three sites (CONSORT flow chart; Figure 1). More details on reasons for non-eligibility are provided in Appendix 1.

Of the LF cohort patients, 107/131 (82%) were nursed with the sheets for all (n=74) or part (n=33) of their inpatient stay. All patients using sheets for only part of their stay were inpatients for 6 days or longer and many returned to normal nursing after an initial period on the sheets. The reasons for 24 patients not being nursed on sheets included: surgeon preference (7/24: 29%) (this included for example patients with unstable fracture required to stay still, complicated burn location or concurrent illness), unplanned overnight stays as patients booked as day cases and not fit to be discharged (5/24: 21%), exclusion following risk assessment (4/24: 17%), no staff availability (3/24: 13%), staff forgetting (1/24: 4%), patient nursed in intensive care unit (1/24: 4%) and no explanation provided (3/24: 12%).

For peer review only - http://bmjopen.bmj.com/site/about/guidelines.xhtml

Proof of concept

Comparison of cohorts at admission

Only one demographic difference was found between the two cohorts at admission (Table 1). A higher proportion of patients receiving antibiotics peri-operatively for graft surgery was reported in the comparator cohort compared to the LF cohort.

For peer review only

Table 1. Demographic differences between comparator (standard care) cohort and low friction (LF) cohort.

	Comparator cohort (N tot=90)	LF cohort (N tot=131)
Centre (Proportion [%])		
A	12/90 [13]	27/131 [21]
B	33/90 [37]	51/131 [39]
C	45/90 [50]	53/131 [40]
Ethnicity (Proportion white British [%])	77/90 [86]	106/131 [81]
Age in years (median [IQR])	48 [23 – 62.5] N=88	41 [15 – 64] N=131
TBSA (median [IQR])	3.3 [1.5 – 7.5] N=90	4 [1.5 – 10.0] N=131
Gender (Proportion male [%])	53/90 [59]	78/131 [60]
Burn type (Proportion scalds [%])	38/90 [42]	37/131 [28]
Location (Proportion [%])		
Head and neck	24/90 [27]	40/131 [31]
Anterior chest	23/90 [26]	34/131 [26]
Posterior chest or back	13/90 [14]	23/131 [18]
Upper limb	29/90 [32]	48/131 [37]
Hand or wrist	21/90 [23]	31/131 [24]
Abdomen	8/90 [9]	18/131 [14]
Buttocks	10/90 [11]	9/131 [7]
Perineum	6/90 [7]	2/131 [2]
Lower limb	31/90 [34]	63/131 [48]
Foot or ankle	16/90 [17]	26/131 [20]
Significant comorbidity (Proportion yes [%])	32/89 [36]	36/131 [27]
ASA grade (Proportion normal healthy patient [%])	45/89 [51]	59/131 [45]
Weight of patient (median [IQR])	69.5 [58.7 – 81.5] N=90	71.8 [55.3 – 86.0] N=131
**Peri-operative antibiotics (Proportion yes [%])***	**73/90 [81]**	**85/131 [65]**
Time to first graft in days (median [IQR])	1 [1 – 5] N=90	2 [0 – 4] N=131

* Highlighted in bold shows 95% confidence interval not crossing 1 in two-sample proportion tests.

Baseline demographic data were also analysed for the subset of nursed vs. not nursed on low friction sheets within the LF cohort. The only differences found between the two subsets were that patients in the LF cohort not nursed on sheets had fewer posterior chest or back burns and a smaller proportion had antibiotics peri-operatively for the first graft (Table 2).

For peer review only

Table 2. Demographic differences between those nursed and not nursed on low friction sheets within the low friction (LF) cohort.

	On sheets (N tot=107)	Not on sheets (N tot=24)
Centre (Proportion [%])		
A	22/107 [21]	5/24 [21]
B	39/107 [37]	12/24 [50]
C	46/107 [43]	7/24 [29]
Ethnicity (Proportion white British [%])	90/107 [84]	16/24 [67]
Age in years (median [IQR])	41 [15 – 64] N=107	42 [20 – 61] N=24
TBSA (median [IQR])	4 [1.5 – 10.0] N=107	2.5 [1 – 7.0] N=24
Gender (Proportion male [%])	64/107 [60]	14/24 [58]
Burn type (Proportion scalds [%])	33/107 [31]	4/24 [17]
Location (Proportion [%])		
Head and neck	34/107 [32]	6/24 [25]
Anterior chest	29/107 [27]	5/24 [21]
Posterior chest or back	**23/107 [22]**	**0/24 [0]**
Upper limb	39/107 [36]	9/24 [38]
Hand or wrist	23/107 [22]	8/24 [33]
Abdomen	15/107 [14]	3/24 [13]
Buttocks	6/107 [6]	3/24 [13]
Perineum	2/107 [2]	0/24 [0]
Lower limb	52/107 [49]	11/24 [46]
Foot or ankle	22/107 [21]	4/24 [17]
Significant comorbidity (Proportion yes [%])	25/107 [23]	11/24 [46]
ASA grade (Proportion normal healthy patient [%])	50/107 [47]	9/24 [38]
Weight of patient (median [IQR])	71.8 [54.2 – 86.0] N=107	71.5 [60.0 – 85.9] N=24
**Peri-operative antibiotics (Proportion yes [%])**	**75/107 [70]**	**10/24 [42]**
Time to first graft in days (median [IQR])	1 [0 – 4] N=107	2 [1 – 5] N=24

[*] Highlighted in bold shows 95% confidence interval not crossing 1 in two-sample proportion tests.

Primary outcome

Data for the primary outcome of graft loss requiring re-grafting was 100% complete in both the LF and comparator cohort. Graft loss requiring re-grafting was 6.7% (6/90) with standard care and 3.8% (5/131) with LF nursing. The odds ratio for this difference was 0.56 but 95% confidence interval crossed 1 (Table 3).

Secondary outcomes

Percentage graft loss

Data for the secondary outcome of percentage graft loss was 100% complete in both cohorts. A larger proportion of patients in the comparator cohort (40%) reported any graft loss compared to the LF cohort (15%) with an odds ratio of 0.27 (95% CI: 0.14, 0.51) (Table 3). Similar patterns were found when graft loss was measured as >5% or >10% reported loss (Table 3).

Length of stay

Two individuals in each cohort had missing data on this outcome. There was no difference between cohorts in any of the hospital LoS measures (Table 3).

For peer review only

Table 3. Proof-of-concept outcomes in the comparator cohort compared to the low friction (LF) cohort.

	Comparator cohort (N tot=90)	LF cohort (N tot=131)	ES [95% CI]*
Primary outcome			
Re-grafted (Proportion yes [%])	6/90 [6.7]	5/131 [3.8]	0.56 [0.16, 1.88]
Secondary outcomes			
Reported graft loss			
**Proportion >0% [%]**	**36/90 [40]**	**20/131 [15]**	**0.27 [0.14, 0.51]**
**Proportion >5% [%]**	**22/90 [24]**	**15/131 [11]**	**0.40 [0.19, 0.82]**
**Proportion >10% [%]**	**15/90 [17]**	**9/131 (7)**	**0.37 [0.15, 0.88]**
Length of stay			
First inpatient length of stay			
median [IQR]	6 [3-13]	7 [3-16]	0 [-2.00, 1.00]**
mean [SD]†	11.5 [15.4]	12.2 [14.7]	-0.64 [-4.69, 3.40]
First inpatient length of stay/% BSA			
median [IQR]	2 [0.9-4]	2 [1.2-3.3]	0 [-0.42, 0.50]**
Total burn service length of stay			
median [IQR]	33.5 [25.5-69.5] N=88	32 [18-51] N=129	5[-2.00, 12.00]**

* ES = Effect Size. For re-graft and graft loss, odds ratios and 95% confidence intervals (CI) are reported. For length of stay, median and mean differences with 95% CI are reported. Highlighted in bold shows 95% CI not crossing 1. Comparator cohort used as control cohort.

** Confidence intervals for generalized Hodges-Lehmann median differences.

† Used for cost analysis.

Economic analyses

Across the three hospital sites, the total purchase costs for the low-friction items were £14,222 and staff training costs were £720. There were no additional charges to launder LF items. This equates to a mean intervention study cost of approximately £114 based on the 131 patients nursed in the low-friction environment. The mean LoS cost (SD) associated with the comparator cohort was £9,608 (12,803) and £10,747 (14,365) for the LF cohort, with a mean difference of £-1,339 (-4,829 to 2,551).

For peer review only

Feasibility outcomes

Practicality

In semi-structured qualitative interviews, staff reported that putting the sheets on the bed was difficult as they became untucked due to the lack of 'fitted' design. They also found that the LF sheets were more difficult to keep clean and dry as the sheets were less absorbent and there was increased pooling due to the nature of burn wounds. However, the slipperiness of the sheets made it easier to move patients in bed. There were also some laundry issues due to the staining of the LF sheets, and sheet snagging. One site had initial issues with considerable laundry sheet loss, but all these issues were resolved and did not hamper the intervention. More detailed findings from staff and patient interviews about the sheets will be reported elsewhere (unpublished data).

Safety

No tissue viability incidents were reported for any patient being nursed on the LF bedding and no surgical site infections were reported. There was also no difference between cohorts in the proportion of patients reporting a positive wound swab as a measure of infection (comparator: 71/87 [81.6%]; LF: 103/131 [78.6%]). Each site reported one patient fall in the LF cohort; two were not using the low friction bedding at the time and the third fell whilst suffering a myocardial infarction unrelated to the LF sheeting.

DISCUSSION

In this study, we have provided proof of concept for the use of a LF environment in patients undergoing skin grafts for burn injury. A consistent difference between the two cohorts before and after the introduction of LF bedding was seen across different ways of assessing

For peer review only

partial graft loss. One in four patients with standard care sheets had at least 5% graft loss whereas only one in eight did on the LF sheets. Although the odds of needing a re-grafting operation was halved in the LF cohort, the confidence interval was wide and crossed 1 due to the low prevalence of re-grafting operations in our study. A much larger study would be needed to provide the same effect size with narrow confidence interval if the primary outcome of re-grafting were to be used in a randomised controlled trial (RCT), necessitating the collection of data from over 2000 grafted patients. We showed no difference in length of stay and associated costs between the two cohorts. Practical issues were mostly easy to resolve, and our results show that the LF bedding is safe for acutely burned patients undergoing skin grafts.

Limitations with our study include the choice of a before and after design to test the intervention. Although this provides us with proof of concept data, only a full RCT could provide the best quality evidence regarding effectiveness of the LF bedding and the causality of the differences we observed. We recognise that the potential for bias is inherent in our chosen design and sought to mitigate this by comparing the demographic variables between the two cohorts. We did not find any patient or demographic characteristics which would suggest an alternative explanation for the differences in graft loss that we observed. Another limitation in this study was the lack of standardised reporting of partial graft loss across clinicians and sites. Following established local protocols, some sites were assessing graft loss, and some were assessing percentage healed, with inconsistency between whether it should be assessed as 90% or 95% healed. Consistency of assessment would allow accurate audits to take place allowing outcome success to be compared across sites and within sites which could have a major impact on clinical practice. However, despite this inconsistency the effect size was consistent across the different ways of reporting.

For peer review only

As this type of LF bedding has not previously been used in patients with burns, comparing our results to existing literature is difficult. However, one study did assess the effectiveness of LF garments, made by the same material and company, in reducing skin breakdown in patients with pressure ulcers.[14] This study showed that there was a significant reduction in the proportion of patients who developed pressure ulcers following use of the LF garments. In addition, fewer patients admitted already with ulcers deteriorated when using the LF garments. This study also suggested that the savings associated with preventing skin breakdown outweighed the cost of the products used.[14]

Implications and future research

Skin graft loss after surgery impacts on patients' outcomes and health service costs. Increased scarring due to the delay in healing will affect patients in terms of cosmetic, psychological and functional outcomes.[22] The magnitude of the effect will depend on the size of the graft loss relative to the original graft. Increased healthcare costs will be related to a need for re-operation, increased need for dressings and outpatient management and potential for infection and scar management. Formally assessing clinical effectiveness through a randomised control trial could be problematic. Given the true low rate of re-grafting, the trial would need to be prohibitively large. If the primary outcome was altered to be percentage graft loss, associated costs such as treating increased scarring would be harder to quantify than re-grafting costs (re-operation, increased length of stay). An alternative might be to consider the data provided by this study as sufficient proof of concept to begin a roll out of LF bedding to burns services for use in grafted patients, and to conduct surveillance of this using routinely collected data.

For peer review only

Conclusion

This study has demonstrated that LF bedding is safe to use with burned patients undergoing skin grafting in acute care settings. Using the LF bedding did not prolong any patient's length of stay and any practical issues arising in relation to sheet usage were relatively easy to resolve. The study showed good proof of concept for LF nursing, suggesting partial graft loss could be reduced if a low friction approach was to become standard. The value of mounting a full randomised controlled trial remains in doubt, and economic modelling using value of information methods would be useful before any further decisions are made.

For peer review only

ACKNOWLEDGEMENTS

We would like to thank everyone who has helped with this study. Especially the patients, children and their parents for their time, effort and enthusiasm, the Research and Innovation, Infection Control and Tissue Viability teams at University Hospitals Bristol, North Bristol and Queen Victoria Hospital NHS Trusts, the burn surgeons' multi-disciplinary teams and research nurses at all sites, and the project steering committee. We thank Pam Warren, our patient representative for her invaluable help and active involvement with this project. This paper presents independent research funded by the National Institute for Health Research (NIHR) under its Research for Patient Benefit (RfPB) Programme (Grant Reference No. PB-PG-0214-33003). The views expressed are those of the authors and not necessarily those of the NHS, the NIHR or the Department of Health. The Centre for Children's Burns Research is part of the Burns Collective, a Scar Free Foundation initiative with additional funding from the Vocational Training Charitable Trust VTCT and Health and Care Research Wales. The

views expressed are those of the authors, and not necessarily those of the Scar Free Foundation or other funding bodies.

REFERENCES

1 Atiyeh BS, Hayek SN, and Gunn W. 2005. New technologies for burn wound closure and healing—review of the literature. *Burns* 31; 944–956.

2 Janžekovic Z. 1970. A new concept in the early excision and immediate grafting of burns. *Journal of Trauma- Injury Infection & Critical Care* 10; 1103–1108.

3 Ong YS, Samuel M, and Song C. 2006. Meta-analysis of early excision of burns. *Burns* 32; 145–150.

4 Cubison TCS, Pape SA, and Parkhouse N. 2006. Evidence for the link between healing time and the development of hypertrophic scars (HTS) in paediatric burns due to scald. *Burns* 32; 992–999.

5 International Burn Injury database. http://www.ibidb.org/.

6 Llanos S, Danilla S, Barraza C, et al. 2006. Effectiveness of negative pressure closure in the integration of split thickness skin grafts: a randomized, double-masked, controlled trial. *Annals of surgery* 244; 700–705.

7 Forter JL, and Castiglione CL. 2012. Skin Grafting. *Techniques in Orthopaedics* 27; 244–249.

8 Petkar KS. 2011. A prospective randomized controlled trial comparing negative pressure dressing and conventional dressing methods on split-thickness skin grafts in burned patients. *Burns* 37; 925–929.

9 Thorne CHM, Gurtner, GC, Chung K, et al. 2007. *Techniques and principles in plastic surgery*. *Grabb and Smith's Plastic Surgery (sixth ed.)*, Lippincott Williams & Wilkins/A Wolters Kluwer Business. p. 8.

10 Wiechman SA, Patterson DR. 2004. Psychosocial aspects of burn injuries. *BMJ : British Medical Journal* 329:391–393.

For peer review only

11 Andersson AE, Bergh I, Karlsson J, et al. 2010. Patients' experiences of acquiring a deep surgical site infection: an interview study. *American Journal of Infection Control* 38; 711–717.

12 Branski LK, Mittermayr R, Herndon DN, et al. 2011. Fibrin sealant improves graft adherence in a porcine full-thickness burn wound model. *Burns* 37; 1360–1366.

13 Hoeller M, Schintler MV, Pfurscheller K, et al. 2014. A retrospective analysis of securing autologous split-thickness skin grafts with negative pressure wound therapy in paediatric burn patients. *Burns* 40; 1116–1120.

14 Smith G, and Ingram A. 2010. Clinical and cost effectiveness evaluation of low friction and shear garments. *Journal of Wound Care* 19; 535–542.

15 http://www.parafricta.com/. Accessed 22 November 2017.

16 Harris P, Taylor R, Thielke R, et al. 2009. Research electronic data capture (REDCap) - A metadata-driven methodology and workflow process for providing translational research informatics support. *Journal of Biomedical Information* 42; 377–381.

17 StataCorp. Stata Statistical Software: Release 14. In. College Station, TX: StataCorp LP; 2015.

18 American Society of Anesthesiologists. ASA physical status classification system. Available at: https://www.asahq.org/resources/clinical-information/asa-physical-status-classification-system.

19 Curtis L. Unit Costs of Health and Social Care, PSSRU. Available from: http://www.pssru.ac.uk/project-pages/unit-costs/2015.

20 Department of Health. National schedule of reference costs for NHS trusts and NHS foundation trusts 2014-2015. Available at: https://www.gov.uk/government/publications/nhs-reference-costs-2014-to-2015.

21 Efron B and Tibshirani RJ. 1993. An introduction to the bootstrap. Chapman and Hall New York.

For peer review only

22 Cubison TCS, Pape SA and Parkhouse N. 2006. Evidence for the link between healing time and the development of hypertrophic scars (HTS) in paediatric burns due to scald injury. *Burns* 32; 992–999.

FIGURE LEGEND

Figure 1. Consort 2010 Flow Diagram for study.

For peer review only

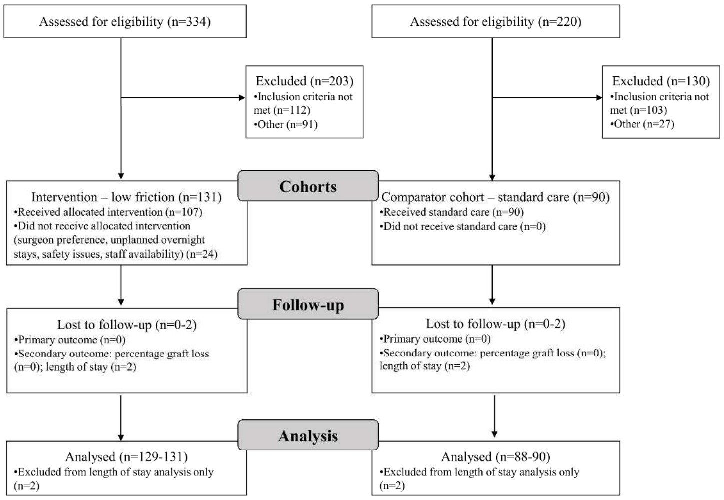

338x190mm (300 x 300 DPI)

Appendix 1. Participant flow to questionnaires and interviews looking at resource use and patient views of the low friction intervention.

334 screened and assessed for eligibility for low friction nursing over 15 months

Not eligible: n=203 (61%)

- Day case only (no overnight stay): 51 (25%)
- Ventilated or on inotropes: 4 (2%)
- VAC dressings were used to maintain grafts: 3 (1.5%)
- Allograft: 6 (3%)
- Not burns: 19 (9%)
- Not skin grafted/surgical plan altered: 24 (12%)
- Missed by research/ward staff: 5 (2%)
- Ineligibility details unavailable: 91 (45%)

131 eligible for low friction nursing cohort (39%)

Not approached for consent to questionnaires/interview: n = 59 (45%)

- No research staff availability: 15 (25%)
- Patient mental health issues: 13 (22%)
- Clinical staff request: 8 (14%)
- Originally day case – consent not sought 5 (8%)
- Non-English speaking: 2 (3%)
- Child protection issues: 2 (3%)
- Parents injured at same time: 1 (2%)
- Other: 13 (22%)

72 patients or parents of patients approached for consent (55%)

Did not consent: n = 20 (28%)

- No research staff availability: 1 (5%)
- Patient mental health issues: 1 (5%)
- Literacy issues: 2 (10%)
- High levels of stress around injury: 4 (20%)
- Patient/carer not interested: 2 (10%)
- No reason given: 6 (30%)
- Other: 4 (20%)

52 (72%) consented to questionnaires and 49 (68%) consented to interview

48 completed at least one questionnaire (92%)
32 participants completed all three questionnaires (62%)
17 interviews carried out (35%)

For peer review only - http://bmjopen.bmj.com/site/about/guidelines.xhtml

BMJ Open

The SILKIE (Skin graftIng Low friKtIon Environment) study: a non-randomised proof-of-concept and feasibility study on the impact of low friction nursing environment on skin grafting success rates in adult and paediatric burns.

Journal:	*BMJ Open*
Manuscript ID	bmjopen-2018-021886.R1
Article Type:	Research
Date Submitted by the Author:	28-Mar-2018
Complete List of Authors:	Hollen, Linda; The Scar Free Foundation Centre for Children's Burn Research, University Hospitals Bristol NHS Foundation Trust; Bristol Medical School, University of Bristol, Centre for Child and Adolescent Health<br>Greenwood, Rosemary; University Hospitals Bristol NHS Foundation Trust, Research and Design Service<br>Kandiyali, Rebecca; The Scar Free Foundation Centre for Children's Burn Research, University Hospitals Bristol NHS Foundation Trust; Bristol Medical School, University of Bristol, Centre for Child and Adolescent Health<br>Ingram, Jenny; Bristol Medical School, University of Bristol, Centre for Child and Adolescent Health<br>Foy, Chris; Gloucester RDSU<br>George, Susan; The Scar Free Foundation Centre for Children's Burn Research, University Hospitals Bristol NHS Foundation Trust<br>Mulligan, Sandra`; The Scar Free Foundation Centre for Children's Burn Research, University Hospitals Bristol NHS Foundation Trust<br>Spickett-Jones, Francesca; The Scar Free Foundation Centre for Children's Burn Research, University Hospitals Bristol NHS Foundation Trust<br>Booth, Simon; Queen Victoria Hospital NHS Foundation Trust, McIndoe Burn Centre<br>Sack, Anthony ; North Bristol NHS Trust, Southmead Hospital<br>Emond, Alan; The Scar Free Foundation Centre for Children's Burn Research, University Hospitals Bristol NHS Foundation Trust; Bristol Medical School, University of Bristol, Centre for Child and Adolescent Health<br>Young, Amber; The Scar Free Foundation Centre for Children's Burn Research, University Hospitals Bristol NHS Foundation Trust
**Primary Subject Heading**:	Evidence based practice
Secondary Subject Heading:	Evidence based practice, Medical management, Public health
Keywords:	skin grafting, low friction, burn injuries, graft loss, burns

SCHOLARONE™
Manuscripts

For peer review only

The SILKIE (Skin graftIng Low friKtIon Environment) study: a non-randomised proof-of-concept and feasibility study on the impact of low friction nursing environment on skin grafting success rates in adult and paediatric burns.

Linda Hollén[1,2], Rosemary Greenwood[3], Rebecca Kandiyali[1,2], Jenny Ingram[2], Chris Foy[4], Susan George[1], Sandra Mulligan[1], Spickett-Jones Francesca[1], Booth Simon[5], Sack Anthony[6], Alan Emond[1,2], Amber Young[1]

[1] The Scar Free Foundation Centre for Children's Burn Research, Bristol Royal Hospital for Children, University Hospitals Bristol NHS Foundation Trust, Bristol, UK.

[2] Centre for Child and Adolescent Health, Bristol Medical School, University of Bristol, Oakfield House, Oakfield Grove, Bristol, UK.

[3] Department of Research and Innovation, University of Hospitals Bristol NHS Foundation Trust, Bristol, UK.

[4] Gloucestershire Research Support Service, Gloucestershire Hospitals NHS Foundation Trust, Gloucester, UK.

[5] McIndoe Burns Centre Queen Victoria Hospital NHS foundation Trust, East Grinstead, UK.

[6] North Bristol NHS Trust, Southmead Hospital, UK.

Correspondence to: Dr Amber Young, email: amber.young1@nhs.net

For peer review only

ABSTRACT

Objectives: To evaluate the impact of low friction (LF) bedding on graft loss in an acute burn care setting, and to examine the feasibility, and costs of using LF bedding compared to standard care.

Design: Proof of concept before and after study with feasibility of delivering the intervention.

Setting: Three burns services within two UK hospital trusts.

Participants: Inclusion criteria were patients older than four weeks, who received a skin graft after burn injury and were admitted overnight. The comparator cohort were eligible patients admitted in a 12-month period before the intervention.

Intervention: Introduction of LF sheets and pillowcases during a 15-month period.

Outcome measures: For proof of concept, the LF and comparator cohorts were compared in terms of number of re-grafting operations (primary), percentage graft loss, average hospital length of stay (LoS), and LoS cost (secondary). Feasibility outcomes were practicality and safety of using LF bedding.

Results: 131 patients were eligible for the LF cohort and 90 patients for the comparator cohort. Although the primary outcome of the proportion needing re-grafting was halved in the LF cohort, the confidence interval crossed 1 (OR [95% CI]: 0.56 [0.16, 1.88]). Partial graft loss (any loss) was significantly reduced in the LF cohort (0.27 [0.14, 0.51]). Average inpatient LoS was no different between the two cohorts (difference in median days [95% CI]: 0 [-2, 1]), and the estimated difference in LoS cost was £-1,139 [-4,829 to 2,551]. Practical issues were easily resolved, and no safety incidents occurred while patients were nursed on LF bedding.

Conclusions: LF bedding is safe to use in burned patients with skin grafts and we have shown proof of concept for the intervention. Further economic modelling is required to see if an appropriately powered randomised control trial would be worthwhile or if roll out across the NHS is justified.

Trial registration: ISRCTN82599687

Keywords: skin grafting, low friction, burn injuries, graft loss, burns

For peer review only

Article Summary

Strengths and limitations of this study

Strengths

- A multi-centre study confirming the feasibility, practicality and safety of using low friction bedding in children and adults with skin grafts.
- Proof of concept that graft loss could be lower in a low friction nursing environment has been demonstrated.

Limitations

- A before and after design restricted the strength of evidence of the effectiveness of the intervention.
- Causality of using a low friction environment for preventing graft loss could not be demonstrated.
- Lack of standardised reporting of partial graft loss limited conclusions of the benefit of the low friction environment.

For peer review only

Contributors: LH performed the analysis of data, contributed to development of methods, interpretation of results, and to the drafting and writing of the manuscript. RG led on development of methods, drafted the research protocol, interpretation of results and to critical reviewing of the manuscript. RK performed the costing analysis, contributed to development of methods, interpretation of results and to critical reviewing of the manuscript. JI contributed to development of qualitative methods, interpretation of results and critical reviewing of the manuscript. CF contributed advice on the costing analysis, contributed to development of methods, interpretation of results and to critical reviewing of the manuscript. SG, SM, FSJ, SB and AS collaborated to develop and lead the intervention, contributed to acquisition of data, interpretation of results and to critical reviewing of the manuscript. AE contributed to development of methods, interpretation of results, consensus meetings and to critical reviewing of the manuscript. AY conceived and led the study and initiated development of the intervention, contributed to development of methods, interpretation of results and to critical reviewing of the manuscript. All authors contributed to and approved the final version and agree to be accountable for all aspects of the work.

Funding: This work was supported by the National Institute for Health Research (NIHR) under its Research for Patient Benefit (RfPB) Programme (Grant Reference No. PB-PG-0214-33003). The views expressed are those of the authors and not necessarily those of the NHS, the NIHR or the Department of Health.

Competing interests: None declared.

Ethics approval: This study had the ethical approval from Wales Research Ethics Committee4 Wrexham (REC reference: 15/WA/0156).

Provenance and peer review: Not commissioned; externally peer reviewed.

Data sharing statement: No additional data available.

For peer review only

INTRODUCTION

Achieving wound closure early after burn injury results in improved survival, better cosmetic outcomes and shorter lengths of hospital stay.[1] To assist wound healing, surgical approaches with early tangential excision and wound closure using skin grafting, are increasingly being applied to deep dermal or full thickness burns.[2,3] Skin grafts are also used in partial thickness burns failing to heal within two to three weeks.[4] There are roughly 1,000 skin grafts undertaken in burns patients annually in England and Wales; 75% undertaken in adults and 25% in children.[5] Integration of grafts to the wound requires vascular and lymphatic revascularization and re-innervation.[6] Healing and 'take' depend upon a well-vascularized clean recipient site, close apposition of the graft to the wound bed and appropriate immobilization of the graft to allow development of new vessels.[7] Sub-optimal take (full or partial graft loss) will delay healing, increase hospital stay, and may also require repeat surgery with increased pain and the potential for infection and increased scar formation with poor cosmetic outcomes.[8,9] These suboptimal outcomes cause distress to patients and impact negatively on physical, social, emotional and economic aspects of their life.[10,11]

Loss of the graft typically occurs during the first few days and may be due to loss of contact with the wound bed due to haematoma, infection or shear.[7,8] It is thought that friction between the dressing and the environment can cause a shearing stress on newly transplanted skin cells and detachment from the wound bed. An even distribution of constant pressure on the graft is therefore likely to increase the chances of successful take.[12] However, maintaining stability and attachment of the graft to the wound-bed can be difficult especially in mobile or semi-mobile patients including children.[13] Low friction (LF) products have been shown to be clinically and cost effective in the prevention of skin breakdown in other at-risk patients[14], but not in patients with burns. LF products are currently in use in the National Health Service

For peer review only

(NHS) in England for premature newborn babies, elderly patients and patients with neurological conditions to prevent pressure ulcers.[15]

We undertook this study using a before and after design to investigate whether a LF nursing environment, using bed sheets and pillow cases (LF [intervention] cohort), could improve skin grafting success rates in adult and paediatric patients with burns compared to standard care using normal bed sheets and pillow cases (comparator cohort). The main aims of this study were: (i) to assess primary and secondary outcome measures around graft loss to provide proof of concept for the use of this technology, (ii) to examine the feasibility of our outcome measures and the practicality and safety of using a LF nursing environment in an acute burn care setting. We also assessed costs associated with the intervention (the purchase, maintenance and laundry of LF items; staff training in the use of LF items) and compared the two cohorts in terms of costs associated with inpatient length of stay (LoS).

METHODS

Study design

We used a non-randomised multicentre study to investigate the feasibility of our intervention. As part of this design, we undertook a before-and-after notes review comparing two cohorts: a low friction (LF) cohort (intervention) and a comparator cohort (standard care), to provide proof of concept through the confidence intervals around effect size.

For peer review only

Participants and data

For both the LF and comparator cohort, patients of more than four weeks of age with burns who received a skin graft and were admitted overnight were assessed for inclusion. Patients were excluded if they were on a ventilator, needing inotropic support or required a Vacuum Assisted Closure (VAC) dressing. Bedding was the only difference between standard care and the LF nursing environment in the form of low friction sheets, cot sheets and pillowcases. Standardised operating procedures were written for the use of these materials both in the bed, in chairs and on equipment used to elevate the wounds. The sheets used were already on the market and available to the NHS.[15] They are made from a synthetic fabric similar to parachute silk, but washable at high temperatures and re-usable. The sheets had a large central panel of the low friction material with a 20.4-inch poly cotton border to the sides and 24.5-inch border top and bottom. The border was to reduce the likelihood of the sheets coming untucked as well as reducing the risk of sliding when patients got out of bed. The pillowcases had low friction material on the top and polycotton on the bottom (to reduce the frequency of pillows sliding down the bed). The ultra-low friction fabrics move freely and smoothly over patients' skin and the underlying surface. The sheets conformed to hospital infection control protocols. For the intervention, we expected a three-month adjustment period, after which the standardised operating procedures around LF environment would be finalised. However, as no changes were made during this time, 15 months of data were collected after the introduction of the LF environment. In participating units, the LF environment became standard care, allowing all eligible patients to be included in the intervention cohort. Clinicians could withdraw patients from the LF nursing if it was felt not to be in the best interest of their patient; however, the patient remained in the cohort to prevent biasing the comparison with the comparator cohort (intention to treat analysis). The comparator cohort data were collected for patients from a 12-month period prior to the

introduction of LF bedding. Comparator patients were identified from theatre record books, the national burn injury database (iBID)[11] and Trust information systems.

Data were collected in three burns services within two hospital trusts; one serving an adult population, one a paediatric population and the third both adult and paediatric patients. The same case report form (CRF) was used in both cohorts. Patients in the LF cohort were invited, by the site research nurse, to consent to complete questionnaires on quality of life and resource use and to take part in a telephone interview within two weeks of being discharged from hospital to explore their views of the LF bedding. This study had the ethical approval from Wales Research Ethics Committee4 Wrexham (REC reference: 15/WA/0156).

Outcomes

Primary proof-of-concept outcome

The primary outcome was skin graft failure rate, as assessed by the proportion of patients who underwent re-grafting surgery.

Secondary proof-of-concept outcomes

We assessed two secondary outcomes: percentage graft loss and length of hospital stay. The method of recording percentage graft loss varied between centres but was recorded as either the % graft failure, $\geq$ 90% healed or $\geq$ 95% healed. For the purposes of estimating % graft loss, we assumed that a graft that was 90% or 95% healed had less than 10% or 5% loss respectively. Length of hospital stay (LoS) was recorded as the first inpatient LoS (total and per % body surface area [BSA]) and total burn service LoS including repeat admissions.

For peer review only - http://bmjopen.bmj.com/site/about/guidelines.xhtml

Feasibility outcomes

We assessed two feasibility outcomes (recorded only in the LF cohort): practicality and safety. The practicality outcome was assessed through staff focus groups or telephone interviews, where any difficulties with bedding use on the wards, laundry problems, and issues with absorbency and staining were discussed. In terms of safety outcomes, we collected data on falls or slips and wound infection. Formal risk assessments were carried out as required by each site for manual handling, tissue viability and infection control. The site leads received all safety incident reports for the designated clinical areas in "real time". Background data regarding the numbers of safety incidents were also known to the site leads. To minimise the risk of falls and prevent slipping, the single bed sheets have a low friction sleeping surface with standard sheeting material around the edge of the bed. Further safety improvements included using neonatal cot sheets as shawls to cradle small children instead of full sized sheets to reduce tripping hazards, and the provision of additional pillow cases to facilitate use on equipment rather than full sized sheets. We used two measures of infection: presence of surgical site infections and wound infection as assessed through wound swab results.

Expected sample size

The overall recruitment figure to the LF cohort was dependent on the number of patients who presented with burn injuries requiring skin grafting and an overnight stay at the three study sites during the recruitment period. This was estimated before study start from the national burn database (iBID).[11] A figure of 200 over the recruitment period was initially predicted based on data from the previous five years. After detailed discussion with the database chair however, it became apparent that the data provided from the database were for individual

For peer review only

graft events, not patients, i.e. 24% of grafts were multiple procedures in single patients. Expected recruitment numbers were therefore revised to 75% of 200, i.e. 150 patients.

Missing data/data quality

All data were anonymised and entered at each site onto a REDCap (Research Electronic Data Capture) database.[16] Initially, all sites used double data entry for input to REDCap and data were checked using the REDCap data comparison facility. Where there were discrepancies, the CRF was checked and the REDCap record amended to contain only correct data. A set of rules was agreed by the management team and applied to subsequent data entry which reduced the error rate and all sites moved to single data entry. Missing data and error checking was continued throughout the study by checking all REDCap records against the CRF for every participant.

Statistical Analyses

Analyses were by intention to treat and performed in Stata v.14.[17] We compared the two cohorts at admission for several demographic and patient characteristic variables: centre admitted to, ethnicity, age, TBSA (total body surface area), gender, burn type, body location burnt, comorbidity present, ASA (American Society of Anesthesiology) grade (physical status classification)[18], body weight, peri-operative antibiotics given, and time to first grafting procedure. Summary statistics used were proportions for categorical variables, and medians and inter quartile range (IQR) or means and standard deviations (SD) for continuous variables. We used two-sample proportion tests to compare the proportion of all categorical demographic and patient characteristic and outcome variables in the two cohorts. Odds ratios and 95% confidence intervals (CI) are reported as a measure of effect size for differences in primary and secondary graft loss outcomes. For LoS outcomes, differences in medians and means with 95% CI are reported.

For peer review only

Economic analyses

The economic work sought to provide information (mean costs and confidence intervals, where appropriate) on the acute health care resource use and costs associated with the new technology, with resources being valued using hospital price information, and national sources of unit costs for staff time and bed days.[19,20] Resource use data collected focussed on: i. the additional costs associated with the intervention (i.e. the purchase, maintenance and laundry of low friction items; staff training in the use of low friction items); data collection on this element was limited to the LF cohort alone and ii. before and after comparison of inpatient LoS. In our analysis based on reference costs[20], we multiplied the cost of an "inlier" bed day by LoS to estimate inpatient care costs at the level of the patient. To account for the skew in our cost data, we used non-parametric bootstrap methods to construct the confidence interval around the incremental inpatient bed day cost.[21]

Patient and public involvement

Patients and the public have been actively involved at all stages of this study. Early work was undertaken before study start to understand the views of patients with burns undergoing skin grafts. Parent and staff views on the sheet material were also sought ahead of the study at Bristol Royal Hospital for Children and further views were asked during a public engagement event midway through the study. Eleven children (age range 11-17yrs) were involved in developing age-appropriate patient consent and information literature through an established young people's advisory group. Patient and public views on further study patient documentation such as adult information sheet, parent information sheet and the consent forms, were obtained from individuals who contacted the Burns Research Centre in Bristol

For peer review

via the 'People in Research' NIHR website. Four members of the public also read and edited the adult patient documentation, so that the purpose of the project was clearer. Feedback on research design and outcome measures obtained was incorporated into the final study documents. Ms Pam Warren, Ambassador for the Scar Free Foundation and burns survivor, read and edited the study protocol and advised regarding patient and public input. She was also involved in key decision-making through her membership and attendance in person at the steering group meetings. Patients were not actively involved in the recruitment to and conduct of the study. Following publication of results, we will disseminate to burn patient groups and the public through plain English written documents.

RESULTS

Participant flow, recruitment and compliance

Recruitment to the low friction (LF) cohort started on 6th October 2015 and was completed on 31st December 2016 – a 65-week period. Of the 334 patients screened for eligibility in the LF cohort, 131 (39%) were eligible for low friction nursing. For the comparator cohort, 90 eligible patients were identified across all three sites (CONSORT flow chart; Figure 1). More details on reasons for non-eligibility are provided in Appendix 1.

Of the LF cohort patients, 107/131 (82%) were nursed with the sheets for all (n=74) or part (n=33) of their inpatient stay. All patients using sheets for only part of their stay were inpatients for 6 days or longer and many returned to normal nursing after an initial period on the sheets. The reasons for 24 patients not being nursed on sheets included: surgeon preference (7/24: 29%) (this included for example patients with unstable fracture required to stay still, complicated burn location or concurrent illness), unplanned overnight stays as patients booked as day cases and not fit to be discharged (5/24: 21%), exclusion following

For peer review only

risk assessment (4/24: 17%), no staff availability (3/24: 13%), staff forgetting (1/24: 4%), patient nursed in intensive care unit (1/24: 4%) and no explanation provided (3/24: 12%).

Proof of concept

Comparison of cohorts at admission

Only one demographic difference was found between the two cohorts at admission (Table 1). A higher proportion of patients receiving antibiotics peri-operatively for graft surgery was reported in the comparator cohort compared to the LF cohort.

Table 1. Demographic differences between comparator (standard care) cohort and low friction (LF) cohort.

	Comparator cohort (N tot=90)	LF cohort (N tot=131)
Centre (Proportion [%])		
A	12/90 [13]	**27**/131 **[**21**]**
B	33/90 [37]	51/131 [39]
C	45/90 [50]	53/131 [40]
Ethnicity (Proportion white British [%])	77/90 [86]	106/131 [81]
Age in years (median [IQR])	48 [23 – 62.5] N=88	41 [15 – 64] N=131
TBSA (median [IQR])	3.3 [1.5 – 7.5] N=90	4 [1.5 – 10.0] N=131
Gender (Proportion male [%])	53/90 [59]	78/131 [60]
Burn type (Proportion scalds [%])	38/90 [42]	37/131 [28]
Location (Proportion [%])		
Head and neck	24/90 [27]	40/131 [31]
Anterior chest	23/90 [26]	34/131 [26]
Posterior chest or back	13/90 [14]	23/131 [18]
Upper limb	29/90 [32]	48/131 [37]
Hand or wrist	21/90 [23]	31/131 [24]
Abdomen	8/90 [9]	18/131 [14]
Buttocks	10/90 [11]	9/131 [7]
Perineum	6/90 [7]	2/131 [2]
Lower limb	31/90 [34]	63/131 [48]
Foot or ankle	16/90 [17]	26/131 [20]
Significant comorbidity (Proportion yes [%])	32/89 [36]	36/131 [27]
ASA grade (Proportion normal healthy patient [%])	45/89 [51]	59/131 [45]
Weight of patient (median [IQR])	69.5 [58.7 – 81.5] N=90	71.8 [55.3 – 86.0] N=131
**Peri-operative antibiotics (Proportion yes [%])***	**73/90 [81]**	**85/131 [65]**
Time to first graft in days (median [IQR])	1 [1 – 5] N=90	2 [0 – 4] N=131

* Highlighted in bold shows 95% confidence interval not crossing 1 in two-sample proportion tests.

For peer review only

Baseline demographic data were also analysed for the subset of nursed vs. not nursed on low friction sheets within the LF cohort. The only differences found between the two subsets were that patients in the LF cohort not nursed on sheets had fewer posterior chest or back burns and a smaller proportion had antibiotics peri-operatively for the first graft (Table 2).

For peer review only

Table 2. Demographic differences between those nursed and not nursed on low friction sheets within the low friction (LF) cohort.

	On sheets (N tot=107)	Not on sheets (N tot=24)
Centre (Proportion [%])		
A	22/107 [21]	5/24 [21]
B	39/107 [37]	12/24 [50]
C	46/107 [43]	7/24 [29]
Ethnicity (Proportion white British [%])	90/107 [84]	16/24 [67]
Age in years (median [IQR])	41 [15 – 64] N=107	42 [20 – 61] N=24
TBSA (median [IQR])	4 [1.5 – 10.0] N=107	2.5 [1 – 7.0] N=24
Gender (Proportion male [%])	64/107 [60]	14/24 [58]
Burn type (Proportion scalds [%])	33/107 [31]	4/24 [17]
Location (Proportion [%])		
Head and neck	34/107 [32]	6/24 [25]
Anterior chest	29/107 [27]	5/24 [21]
Posterior chest or back	**23/107 [22]**	**0/24 [0]**
Upper limb	39/107 [36]	9/24 [38]
Hand or wrist	23/107 [22]	8/24 [33]
Abdomen	15/107 [14]	3/24 [13]
Buttocks	6/107 [6]	3/24 [13]
Perineum	2/107 [2]	0/24 [0]
Lower limb	52/107 [49]	11/24 [46]
Foot or ankle	22/107 [21]	4/24 [17]
Significant comorbidity (Proportion yes [%])	25/107 [23]	11/24 [46]
ASA grade (Proportion normal healthy patient [%])	50/107 [47]	9/24 [38]
Weight of patient (median [IQR])	71.8 [54.2 – 86.0] N=107	71.5 [60.0 – 85.9] N=24
**Peri-operative antibiotics (Proportion yes [%])**	**75/107 [70]**	**10/24 [42]**
Time to first graft in days (median [IQR])	1 [0 – 4] N=107	2 [1 – 5] N=24

[*] Highlighted in bold shows 95% confidence interval not crossing 1 in two-sample proportion tests.

Primary outcome

Data for the primary outcome of graft loss requiring re-grafting was 100% complete in both the LF and comparator cohort. Graft loss requiring re-grafting was 6.7% (6/90) with standard care and 3.8% (5/131) with LF nursing. The odds ratio for this difference was 0.56 but 95% confidence interval crossed 1 (Table 3).

Secondary outcomes

Percentage graft loss

Data for the secondary outcome of percentage graft loss was 100% complete in both cohorts. A larger proportion of patients in the comparator cohort (40%) reported any graft loss compared to the LF cohort (15%) with an odds ratio of 0.27 (95% CI: 0.14, 0.51) (Table 3). Similar patterns were found when graft loss was measured as >5% or >10% reported loss (Table 3).

Length of stay

Two individuals in each cohort had missing data on this outcome. There was no difference between cohorts in any of the hospital LoS measures (Table 3).

For peer review only

Table 3. Proof-of-concept outcomes in the comparator cohort compared to the low friction (LF) cohort.

	Comparator cohort (N tot=90)	LF cohort (N tot=131)	ES [95% CI]*	P-value**
Primary outcome				
Re-grafted (Proportion yes [%])	6/90 [6.7]	5/131 [3.8]	0.56 [0.16, 1.88]	0.33
Secondary outcomes				
Reported graft loss				
**Proportion >0% [%]**	**36/90 [40]**	**20/131 [15]**	**0.27 [0.14, 0.51]**	**<0.001**
**Proportion >5% [%]**	**22/90 [24]**	**15/131 [11]**	**0.40 [0.19, 0.82]**	**0.01**
**Proportion >10% [%]**	**15/90 [17]**	**9/131 (7)**	**0.37 [0.15, 0.88]**	**0.02**
Length of stay				
First inpatient length of stay (LoS)				
median [IQR]	6 [3-13]	7 [3-16]	0 [-2.00, 1.00] †	0.51
mean [SD]††	11.5 [15.4]	12.2 [14.7]	-0.64 [-4.69, 3.40]	
First inpatient LoS/% BSA				
median [IQR]	2 [0.9-4]	2 [1.2-3.3]	0 [-0.42, 0.50] †	0.87
Total burn service LoS				
median [IQR]	33.5 [25.5-69.5] N=88	32 [18-51] N=129	5[-2.00, 12.00] †	0.12

* ES = Effect Size. For re-graft and graft loss, odds ratios and 95% confidence intervals (CI) are reported. For length of stay, median and mean differences with 95% CI are reported. Highlighted in bold shows 95% CI not crossing 1. Comparator cohort used as control cohort.
** P-values stem from two-sample proportion tests or Mann-Whitney U-tests.
† Confidence intervals for generalized Hodges-Lehmann median differences.
†† Used for cost analysis.

For peer review only

Economic analyses

Across the three hospital sites, the total purchase costs for the low-friction items were £14,222 and staff training costs were £720. There were no additional charges to launder LF items. This equates to a mean intervention study cost of approximately £114 based on the 131 patients nursed in the low-friction environment. The mean LoS cost (SD) associated with the comparator cohort was £9,608 (12,803) and £10,747 (14,365) for the LF cohort, with a mean difference of £-1,339 (-4,829 to 2,551).

Feasibility outcomes

Practicality

In semi-structured qualitative interviews, staff reported that putting the sheets on the bed was difficult as they became untucked due to the lack of 'fitted' design. They also found that the LF sheets were more difficult to keep clean and dry as the sheets were less absorbent and there was increased pooling due to the nature of burn wounds. However, the slipperiness of the sheets made it easier to move patients in bed. There were also some laundry issues due to the staining of the LF sheets, and sheet snagging. One site had initial issues with considerable laundry sheet loss, but all these issues were resolved and did not hamper the intervention. More detailed findings from staff and patient interviews about the sheets is reported elsewhere.[22]

Safety

No tissue viability incidents were reported for any patient being nursed on the LF bedding and no surgical site infections were reported. There was also no difference between cohorts in the proportion of patients reporting a positive wound swab as a measure of infection (comparator: 71/87 [81.6%]; LF: 103/131 [78.6%]). Each site reported one patient fall in the LF cohort; two were not using the low friction bedding at the time and the third fell whilst suffering a myocardial infarction unrelated to the LF sheeting.

DISCUSSION

In this study, we have provided proof of concept for the use of a LF environment in patients undergoing skin grafts for burn injury. A consistent difference between the two cohorts

For peer review only

before and after the introduction of LF bedding was seen across different ways of assessing partial graft loss. One in four patients with standard care sheets had at least 5% graft loss whereas only one in eight did on the LF sheets. Although the odds of needing a re-grafting operation was halved in the LF cohort, the confidence interval was wide and crossed 1 due to the low prevalence of re-grafting operations in our study. A much larger study would be needed to provide the same effect size with narrow confidence interval if the primary outcome of re-grafting were to be used in a randomised controlled trial (RCT), necessitating the collection of data from over 2000 grafted patients. We showed no difference in length of stay and associated costs between the two cohorts. Practical issues were mostly easy to resolve, and our results show that the LF bedding is safe for acutely burned patients undergoing skin grafts.

Limitations with our study include the choice of a before and after design to test the intervention. Although this provides us with proof of concept data, only a full RCT could provide the best quality evidence regarding effectiveness of the LF bedding and the causality of the differences we observed. We recognise that the potential for bias is inherent in our chosen design and sought to mitigate this by comparing the demographic variables between the two cohorts. We did not find any patient or demographic characteristics which would suggest an alternative explanation for the differences in graft loss that we observed. Another limitation in this study was the lack of standardised reporting of partial graft loss across clinicians and sites. Following established local protocols, some sites were assessing graft loss, and some were assessing percentage healed, with inconsistency between whether it should be assessed as 90% or 95% healed. Consistency of assessment would allow accurate audits to take place allowing outcome success to be compared across sites and within sites

For peer review only

which could have a major impact on clinical practice. However, despite this inconsistency the effect size was consistent across the different ways of reporting.

As this type of LF bedding has not previously been used in patients with burns, comparing our results to existing literature is difficult. However, one study did assess the effectiveness of LF garments, made by the same material and company, in reducing skin breakdown in patients with pressure ulcers.[14] This study showed that there was a significant reduction in the proportion of patients who developed pressure ulcers following use of the LF garments. In addition, fewer patients admitted already with ulcers deteriorated when using the LF garments. This study also suggested that the savings associated with preventing skin breakdown outweighed the cost of the products used.[14]

Implications and future research

Skin graft loss after surgery impacts on patients' outcomes and health service costs. Increased scarring due to the delay in healing will affect patients in terms of cosmetic, psychological and functional outcomes.[23] The magnitude of the effect will depend on the size of the graft loss relative to the original graft. Increased healthcare costs will be related to a need for re-operation, increased need for dressings and outpatient management and potential for infection and scar management. Formally assessing clinical effectiveness through a randomised control trial could be problematic. Given the true low rate of re-grafting, the trial would need to be prohibitively large. If the primary outcome was altered to be percentage graft loss, associated costs such as treating increased scarring would be harder to quantify than re-grafting costs (re-operation, increased length of stay). An alternative might be to consider the data provided by this study as sufficient proof of concept to begin a roll out of LF bedding to burns services for use in grafted patients, and to conduct surveillance of this using routinely collected data.

For peer review only

Although introducing such a new method into daily nursing may increase workload initially, it is unlikely to be a significant cost-driver. At the time of our study, fitted sheets to fit the hospital beds were not available, so we had to use flat sheets which were more time-consuming for nursing staff. If the sheets were to be adopted and a fitted version found to be acceptable, it is unlikely that additional nursing time would be required.

Conclusion

This study has demonstrated that LF bedding is safe to use with burned patients undergoing skin grafting in acute care settings. Using the LF bedding did not prolong any patient's length of stay and any practical issues arising in relation to sheet usage were relatively easy to resolve. The study showed good proof of concept for LF nursing, suggesting partial graft loss could be reduced if a low friction approach was to become standard. The value for the NHS of mounting a full randomised controlled trial remains in doubt, and economic modelling using value of information methods is currently underway before any further decisions are made.

ACKNOWLEDGEMENTS

We would like to thank everyone who has helped with this study. Especially the patients, children and their parents for their time, effort and enthusiasm, the Research and Innovation, Infection Control and Tissue Viability teams at University Hospitals Bristol, North Bristol and Queen Victoria Hospital NHS Trusts, the burn surgeons' multi-disciplinary teams and research nurses at all sites, and the project steering committee. We thank Pam Warren, our patient representative for her invaluable help and active involvement with this project. This paper presents independent research funded by the National Institute for Health Research

For peer review only

(NIHR) under its Research for Patient Benefit (RfPB) Programme (Grant Reference No. PB-PG-0214-33003). The views expressed are those of the authors and not necessarily those of the NHS, the NIHR or the Department of Health. The Centre for Children's Burns Research is part of the Burns Collective, a Scar Free Foundation initiative with additional funding from the Vocational Training Charitable Trust VTCT and Health and Care Research Wales. The views expressed are those of the authors, and not necessarily those of the Scar Free Foundation or other funding bodies.

For peer review only

REFERENCES

1 Atiyeh BS, Hayek SN, and Gunn W. 2005. New technologies for burn wound closure and healing—review of the literature. *Burns* 31; 944–956.

2 Janžekovic Z. 1970. A new concept in the early excision and immediate grafting of burns. *Journal of Trauma- Injury Infection & Critical Care* 10; 1103–1108.

3 Ong YS, Samuel M, and Song C. 2006. Meta-analysis of early excision of burns. *Burns* 32; 145–150.

4 Cubison TCS, Pape SA, and Parkhouse N. 2006. Evidence for the link between healing time and the development of hypertrophic scars (HTS) in paediatric burns due to scald. *Burns* 32; 992–999.

5 International Burn Injury database. http://www.ibidb.org/.

6 Llanos S, Danilla S, Barraza C, et al. 2006. Effectiveness of negative pressure closure in the integration of split thickness skin grafts: a randomized, double-masked, controlled trial. *Annals of surgery* 244; 700–705.

7 Forter JL, and Castiglione CL. 2012. Skin Grafting. *Techniques in Orthopaedics* 27; 244–249.

8 Petkar KS. 2011. A prospective randomized controlled trial comparing negative pressure dressing and conventional dressing methods on split-thickness skin grafts in burned patients. *Burns* 37; 925–929.

9 Thorne CHM, Gurtner, GC, Chung K, et al. 2007. *Techniques and principles in plastic surgery. Grabb and Smith's Plastic Surgery (sixth ed.)*, Lippincott Williams & Wilkins/A Wolters Kluwer Business. p. 8.

10 Wiechman SA, Patterson DR. 2004. Psychosocial aspects of burn injuries. *BMJ : British Medical Journal* 329:391–393.

11 Andersson AE, Bergh I, Karlsson J, et al. 2010. Patients' experiences of acquiring a deep surgical site infection: an interview study. *American Journal of Infection Control* 38; 711–717.

12 Branski LK, Mittermayr R, Herndon DN, et al. 2011. Fibrin sealant improves graft adherence in a porcine full-thickness burn wound model. *Burns* 37; 1360–1366.

13 Hoeller M, Schintler MV, Pfurscheller K, et al. 2014. A retrospective analysis of securing autologous split-thickness skin grafts with negative pressure wound therapy in paediatric burn patients. *Burns* 40; 1116–1120.

14 Smith G, and Ingram A. 2010. Clinical and cost effectiveness evaluation of low friction and shear garments. *Journal of Wound Care* 19; 535–542.

15 http://www.parafricta.com/. Accessed 22 November 2017.

16 Harris P, Taylor R, Thielke R, et al. 2009. Research electronic data capture (REDCap) - A metadata-driven methodology and workflow process for providing translational research informatics support. *Journal of Biomedical Information* 42; 377–381.

17 StataCorp. Stata Statistical Software: Release 14. In. College Station, TX: StataCorp LP; 2015.

18 American Society of Anesthesiologists. ASA physical status classification system. Available at: https://www.asahq.org/resources/clinical-information/asa-physical-status-classification-system.

For peer review only

19 Curtis L. Unit Costs of Health and Social Care, PSSRU. Available from: http://www.pssru.ac.uk/project-pages/unit-costs/2015.

20 Department of Health. National schedule of reference costs for NHS trusts and NHS foundation trusts 2014-2015. Available at: https://www.gov.uk/government/publications/nhs-reference-costs-2014-to-2015.

21 Efron B and Tibshirani RJ. 1993. An introduction to the bootstrap. Chapman and Hall New York.

22 Whale K, Ingram J, George S, et al. 2018. Exploring the acceptability of using low-friction bedding with burns patients: qualitative results from the SILKIE study. *Burns* (In press).

23 Cubison TCS, Pape SA and Parkhouse N. 2006. Evidence for the link between healing time and the development of hypertrophic scars (HTS) in paediatric burns due to scald injury. *Burns* 32; 992–999.

FIGURE LEGEND

Figure 1. Consort 2010 Flow Diagram for study.

For peer review only

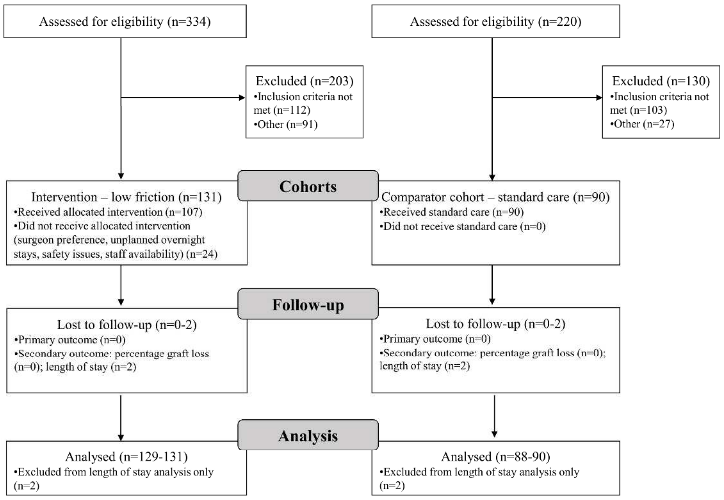

338x190mm (300 x 300 DPI)

review only

Appendix 1. Participant flow to questionnaires and interviews looking at resource use and patient views of the low friction intervention.

334 screened and assessed for eligibility for low friction nursing over 15 months

Not eligible: n=203 (61%)

- Day case only (no overnight stay): 51 (25%)
- Ventilated or on inotropes: 4 (2%)
- VAC dressings were used to maintain grafts: 3 (1.5%)
- Allograft: 6 (3%)
- Not burns: 19 (9%)
- Not skin grafted/surgical plan altered: 24 (12%)
- Missed by research/ward staff: 5 (2%)
- Ineligibility details unavailable: 91 (45%)

131 eligible for low friction nursing cohort (39%)

Not approached for consent to questionnaires/interview: n = 59 (45%)

- No research staff availability: 15 (25%)
- Patient mental health issues: 13 (22%)
- Clinical staff request: 8 (14%)
- Originally day case – consent not sought 5 (8%)
- Non-English speaking: 2 (3%)
- Child protection issues: 2 (3%)
- Parents injured at same time: 1 (2%)
- Other: 13 (22%)

72 patients or parents of patients approached for consent (55%)

Did not consent: n = 20 (28%)

- No research staff availability: 1 (5%)
- Patient mental health issues: 1 (5%)
- Literacy issues: 2 (10%)
- High levels of stress around injury: 4 (20%)
- Patient/carer not interested: 2 (10%)
- No reason given: 6 (30%)
- Other: 4 (20%)

52 (72%) consented to questionnaires and 49 (68%) consented to interview

48 completed at least one questionnaire (92%)
32 participants completed all three questionnaires (62%)
17 interviews carried out (35%)

For peer review only - http://bmjopen.bmj.com/site/about/guidelines.xhtml
